# Memory for the Future: Psychodynamic Approach to Time and Self Through the Default Network

**DOI:** 10.3389/fnhum.2022.885315

**Published:** 2022-06-16

**Authors:** Filippo Cieri

**Affiliations:** Department of Neurology, Cleveland Clinic Lou Ruvo Center for Brain Health, Las Vegas, NV, United States

**Keywords:** memory, time, default network, psychodynamic neuroscience, brain imaging, resting state—fMRI, psychoanalysis

## Abstract

Time exists in us, and our self exists in time. Our self is affected and shaped by time to the point that a better understanding of the former can aid the understanding of the latter. Psychoanalysis works through self and time, where the self is composed of the biopsychosocial history (the past) of the individual and able to map a trajectory for the future. The psychoanalytic relationship starts from a “measurement”: an active process able to alter the system being measured—the self—continuously built over time. This manuscript, starts from the philosophical and scientific tradition of a proximity between time and self, suggesting a neural overlapping at the Default Network. A historical and scientific background will be introduced, proposing a multidisciplinary dimension that has characterized the birth of psychoanalysis (its past), influencing its present and future in the dialogue with physics and neuroscience. After a historical scientific introduction, a neural *entanglement* between past and future at the Default Network level will be proposed, tracing a link with the self at the level of this network. This hypothesis will be supported by studies in cognitive neurosciences and functional neuroimaging which have used the resting state functional Magnetic Resonance Imaging. The ontogenetic development of time perception will be discussed, consistent with self-development and the Default Network’s function. The most common form of dementia, the Alzheimer’s Disease, in which the perception of time is brutally impaired together with a loss of the self’s functions will be proposed to support this idea. Finally, the potential theoretical and clinical significance for psychoanalysis and psychodynamic neurosciences, will be discussed.

*“Who controls the past controls the future: who controls the present controls the past.”*

(*Nineteen Eighty-Four;* Orwell, 1949).

## Introduction

The proximity between time and self has deep philosophical roots. The experience of time is made by the self, where subjective time is associated to the conscious self as an enduring entity over time (Edmund Husserl) and subjective time being in essence embodied (Maurice Merleau-Ponty) (Zahavi, 2005; Wittmann, 2009, 2013). Recent neuroscientific approaches have underlined the affective and interoceptive states of the body as necessary to create a sense of time (Craig, 2009, 2015; Droit-Volet and Gil, 2009; Wittmann et al., 2015).

The renewed discussion of the free energy principle, has given to the *mindbrain* system a greater probabilistic perspective, restarting a dialogue among physics, philosophy, psychology, neuroscience, and psychoanalysis that began with Freud. In this dialogue with neuroscience, neuropsychoanalysis has been accused of using the chemistry of ink to appreciate Van Gogh’s art (Blass and Carmeli, 2007; see Yovell et al., 2015 for a reply to this criticism). In other words, the neuroscientific approach in psychoanalysis has been accused of using the brain’s language to translate the mind’s dimension. Although neuroscience and psychoanalysis have different languages, often studying different aspects of the mindbrain, a dual-aspect monistic position on the philosophical mind-body problem allows for the investigation of the causal mechanism of consciousness not in the manifest brain but rather in its functional organization, which ultimately underpins both the physiological and the psychological manifestations of experience (Cieri and Esposito, 2019). A dual-aspect monism approach will be used to explore the relationship between self and time in this manuscript. Spinoza, Fechner, Schopenhauer, all shared a dual-aspect monistic view, and one of the most intriguing interdisciplinary contributions in this perspective comes from the cooperation between physics and psychoanalysis personified by the collaboration between Wolfgang Pauli and Carl Gustav Jung (see Atmanspacher, 2012). This approach on mental and material problem is strictly associated with ideas and reflections that emerged during the development of quantum theory. The *Pauli-Jung conjecture* had the innovative characteristic of approaching the dual nature (mental and material) in terms of complementarity, a concept itself borrowed from psychology.^1^

Pauli and Jung shared the idea of an unknowable reality, in which the material and the subjective worlds are two complementary manifestations of reality. In a letter to Rosenfeld (1952), Pauli claimed that both quantum physics and the psychology of the unconscious need a symbolic psychophysical unitary language for the invisible reality, and this need was the goal he aspired (von Meyenn, 1996, p. 593; also cited in Alcaro et al., 2017, p. 7).

Cognitive neuroscience uses neuroimaging’ methods to explore brain structures, functions, and their correlations, in which the functional Magnetic Resonance Imaging (fMRI) is a common non-invasive techniques. This manuscript will focus its approach only on this brain imaging method, specifically through the resting state. Although Hameroff and Penrose (2014) call into question a yet-to-be-discovered theory of quantum gravity to understand how the brain is able to work with non-computable functions (in which microtubules should represent the sites of the associated quantum gates), on the other hand Koch and Hepp (2006) claim that neuroscience does not need quantum physics to explain the material basis of consciousness, understandable within a purely neurobiological framework. Certainly, a neurobiological approach is necessary in the investigation of mindbrain functioning, but a dialogue with psychoanalysis can be helpful for neuroscientists (Kandel, 1999, 2016). In this sense, quantum physics can be an equally desirable partner for psychoanalysis and cognitive neurosciences in the study of the mindbrain system, consciousness, and self.

This manuscript is not proposing thoughts, affects, drives, and desires as *pieces of brain*, offering a new form of phrenology or localizationism. The proposal is not even based on quantum physics or neuroscientific methods to reveal the secret of self, because those approaches alone cannot answer all the questions about inner human complexity. Nevertheless, it would be naïve to ignore that it is no longer time for a psychoanalytic *brainless* approach in the investigation of the mind, as much as it would be anachronistic to accept a *mindless* neuroscientific approach currently rich in algorithms and poor in subjectivity. The temporospatial dynamics of the brain’s spontaneous activity shapes individual mental states, the way the subjects experience themselves and others in time and space (Fingelkurts et al., 2010; Northoff, 2011). The predictive brain, the free energy principle, the study of consciousness, the neural networks—with their features of integration and segregation—and the study of brain entropy in neuropsychological functions and dysfunctions, in the past decade have undoubtedly brought new impetus to the dialogue between mind and brain, between psychoanalysis and neuroscience.

*Psychodynamics* is the other name of psychoanalysis, coined by the Freud’s research supervisor at the University of Vienna: Ernst von Brücke. Together with Hermann von Helmholtz and Emil Du Bois-Reymond, von Brücke was one of the founders of the Physical Society in Berlin (Cieri et al., 2021a). von Brücke and von Helmholtz tried to apply thermodynamics to the psyche, treating the human affects and instincts as energy in physical terms. For instance, to describe the “death drive” (*Todestrieb)* (Freud, 1920) used physics, saying that it is a natural direction of the organic matter to go back to its previous inorganic state; this is an “ancestral dissipation” of energy that follows the entropy direction, expressed by the second law of thermodynamics.

Without forgetting the main clinical component of psychoanalysis, the aim of this paper is to show a link between time and self at the level of a specific neural network, retracing the path of the past, to find *a memoire for the future* of psychoanalysis, in its relationship with physics and neuroscience and remembering that one of the reasons why Freud abandoned this dialogue was the lack of materials and methods, we can nowadays instead rely on.

It will be proposed an exploration of our sense of time in a neural network considered crucial for our self: the Default Mode Network (Raichle et al., 2001), also called Default Network (hereafter DN), closely related to our sense of self, or self-consciousness (Qin and Northoff, 2011; Northoff, 2011; Wittmann, 2013; Wittmann et al., 2015; Davey et al., 2016; Northoff, 2016; Cieri and Esposito, 2019), where the individual sense of past and future seems *entangled*. The spontaneous activity within the DN seems associated with remarkable components of human mental life, such as perception of time and self. To support this, the ontological sense of time will be explored, parallel with the growth of DN’s features, limiting the exploration to studies whose authors use the resting state approach, through fMRI. It will also rely on an example from a clinical neuropsychological field, from the most common form of dementia: the Alzheimer Disease (AD), in which the past seems to crumble inexorably without leaving room for any future of the subject. In this form of dementia, the subject’s self is equally crushed. Finally, potential repercussions for the clinical and therapeutic dimension will be discussed.

## Historical and Theoretical Common Ground Between Quantum Physics and Psychoanalysis

Although at a first glance quantum physics can be perceived as far as possible from psychoanalysis, they share some theoretical and symbolic points that might be worth introducing.

A first connection between psychoanalysis and quantum physics is from a theoretical point of view: both disciplines underline the centrality of relationship. Unlike classical physics, in quantum mechanics the value of a variable is given only at its interactions and this value is only relative to the (other) system affected by the interaction. Here “relative” is used in the same sense in which velocity is a property of a system relative to another system in classical mechanics (Laudisa and Rovelli, 2002). In this context, an electron is considered a set of jumps from one interaction to another; therefore it would not have a discrete position, except in relation to something else (Rovelli, 2014). We can find a speculative parallel in psychoanalysis where relationship is a milestone concept, a *Copernican revolution*, both as a vulnerability component for the development of psychological disorders and a *cure* factor through the psychoanalytic relationship. The attachment, as the first form of relationship, builds not only the baby’s relationship mode, or the baby’s personality, but rather it shapes his identity, the very existence, the individual self. There is a large psychoanalytic and neuroscientific literature on attachment, as a relationship par excellence. Although this point is beyond the scope of this manuscript, it is possible at least to cite studies by important authors to endorse the common quantum and psychoanalytic idea that “reality is only interaction” (Rovelli, 2014, p. 29). Among these authors, Spitz (1945) pointed out the negative effects of maternal deprivation in early syndromes such as hospitalism, in which children in orphanages do not even have a maternal figure (no affective relationship at all), reported psychophysical damage that can lead to death. Also, Harlow et al. (1965) and Hofer (1994) have described the central role of attachment through the animal model, while Bion (1962), Bowlby (1958; 1969; 1973), Klein (1948), and Winnicott (1955) have used human model and clinical settings.^2^

**FIGURE 1:**
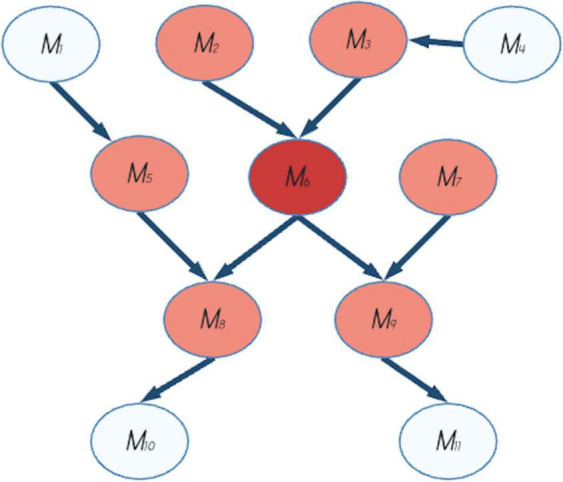
Example of a Markov blanket. The Markov blanket of the node *M6* (in red in the figure) comprises the set of parents, children and spouses of the node and it is indicated by the pink nodes. *M2* and *M3* are the parents, *M8* and *M9* are children, *M5* and *M7* are spouses of *M6*. The other nodes (*M1, M4, M10*, and *M11*) are not in the Markov blanket of *M6*.

From a neuroscientific perspective, relationship among states, neurons and neural networks is a milestone of cognitive neuroscience and the Free Energy Principle (FEP).^3^ One of the key concepts of the FEP is the Markov blanket (see Kirchhoff et al., 2018 for details and Figure 1 for a schematic example). This concept supports the idea that the peculiar function of separation between an internal and an external world allows for relationships between different states. It was born as a statistical method used in machine learning, but its application has been extended further and wider to almost every layer of organization in nature (Clark, 2016; Kirchhoff et al., 2018; Ramstead et al., 2018; Cieri et al., 2021a). In FEP, the Markov blanket divides a system into internal, active, sensory, and external, and without this separation (between states) communication (relationships) would be not possible.

A second aspect in which psychoanalysis and quantum physics can find a communication channel through the lens of psychodynamic neuroscience—the only object of this manuscript—is time. The mystery of time seems to be more about ourselves than about the cosmos; therefore understanding ourselves means reflecting on time, but understanding time means reflecting on ourselves (Wittmann et al., 2015; Rovelli, 2017). In this sense, the current proposal concerns a close neuropsychodynamic relationship between time and self.

Although recognizing the different scales of reality that this manuscript is taking into consideration, comparing psychoanalysis and quantum physics, the attempt will be to find correlations between the human world and the minuteness of the scale at which quantum properties of spacetime (presumably) manifest themselves, recalling a kind of *fractality of reality*, where similar patterns recur at progressively smaller scales. Basically, effects of quantum theory can also appear at larger scales, such as in the case of superconductivity or superfluidity. It is worth mentioning that in 1955 (Pauli, 1955) published a paper on mirror symmetry, expressing a feeling of a deep connections between mind and matter and an inevitable consonance of “inside” and “outside.” In his words:

[…] unconscious motives are always involved thereby. […]. Physics relies on a relation of mirror symmetry between mind and nature (cited in Atmanspacher and Primas, 2006, p. 8).

If, on one hand, general relativity claims that spacetime location is relational only, on the other hand, quantum mechanics tells us that any dynamic entity is subject to Heisenberg’s uncertainty at a small scale. Therefore, we need a relational notion of a quantum spacetime to understand Planck scale physics (Rovelli, 1998). The relational nature of reality is studied through quantum mechanics in its appropriate laboratory scale and by psychoanalysis within a clinical setting.

The discussion around the FEP and the neural networks has created the conditions for a new *royal road* for psychodynamic neuroscience (Cieri and Esposito, 2019). In this bridge the concept of entropy plays a key role. Boltzmann’s work on entropy can be interpreted through a puzzling conclusion: the difference between past and future can be seen as resulting from a blur to which we are doomed. In other words, entropy exists because we describe the world in a blurry way (Rovelli, 2017).

In *A lover’s discourse: fragments* by Barthes and Howard (1978), a child disassembles a watch, to find out what time actually is. Hoping to avoid a similar naivety, in the next sections it will be proposed an attempt to look closer into a specific neural network to see how the past and future are intertwined in our perception of time, and in turn connected with our sense of self. This attempt takes into consideration neuropsychological studies on development, maturation, and impairment of sense of time and self-function through resting state fMRI studies.

## The Resting State

Andreasen et al. (1995) used positron emission tomography (PET) to analyze the neural correlates of random episodic memory, where the uncensored thinking about experience comes to the subject’s mind. These authors pointed out that the ability of the subject to place events in time and to reference them to oneself is at the base of consciousness and self-awareness. They also recalled that the kind of random memory they were investigating is the same used in psychoanalysis through free associations and coined the acronym REST (*Random Episodic Silent Thought*) to indicate this specific form of thinking, now the most used approach in cognitive neuroscience using brain imaging (Cieri et al., 2020). The REST approach comes from the idea to explore the brain’s activity when the subject is not involved in any specific cognitive or motor task, lying still in the scanner, a dimension most likely identical to the one identified by the father of American psychology, James (1890), called *stream of consciousness*, 100 years before the REST condition was investigated by brain imaging. This phase was already applied in imaging studies, but it was treated as a control condition to compare the task state. In other words, the investigation of the brain’s activation during a task (e.g., a motor task) was the experimental state (or task-positive), which needs a control condition, the resting state, or task-negative, emphasizing its nature partly in contrast with the engagement during the tasks. Both during rest and task, the activity of the brain is organized into intrinsic networks (i.e., brain areas that synchronize their activity) (Raichle et al., 2001). The resting state approach describes how the brain *does not rest, especially during REST*.

As mentioned, the idea that our mental life happens mostly unconsciously is not recent; both Freud and James, from a psychological perspective, claimed that most of our perception is unconscious, emphasizing the importance of the emotional, instinctual and drive processes. They were inspired, and they in turn have inspired philosophers, physicists, and neuroscientists.

On one hand, Schopenhauer’s metaphysics had a key role in Freud’s theory. On the other hand, Helmholtz’s thermodynamics had a fundamental place in building the foundations for the current bridge between neuroscience and psychoanalysis. It is worth mentioning the influence of Schopenhauer (1969) from a philosophical perspective, about the nature and the importance of unconscious processes. Schopenhauer (1969) claimed:

Unconsciousness is the original and natural condition of all things, and therefore is also the basis from which, in particular species of beings, consciousness appears as their highest efflorescence; and for this reason, even then unconsciousness still always predominates (p. 142).

On the physics side, the influence of von Brücke and von Helmholtz on the same topic also played a crucial role in psychoanalysis. When Helmholtz was studying the nervous system, it was a common belief that in the central nervous system, the nerve fibers transmit the electrical energy at the speed of light, therefore our visual perception was reputed instantaneous. In 1852, Helmholtz was able to measure this actual speed, discovering that it took about 20 ms for the nerve impulse to travel 1 m (von Helmholtz, 1971; also cited in Frith, 2007, p. 41). Our perception is driven by involuntary, pre-rational, and reflex-like mechanisms as a crucial part of our formation of visual impressions. Helmholtz called this implicit process *unconscious inference*, which has had a terrific impact on psychoanalysis, psychology, and the FEP.

The birth and evolution of psychoanalysis have been marked by the dialogue between these authors coming from their respective disciplines. The psychodynamic approach to neuroscience, also thanks to the application of the resting state, is bringing this dialogue back to the center of theory and research, helping the advancement of knowledge toward the consciousness, the unconscious, and the self.^4^

For the main part, the resting state is implicit, involuntary, unconscious, or subconscious. The individual’s mindbrain system at REST shows a plenty of activity, and midline brain regions show an increase spontaneous activity (Northoff and Panksepp, 2008), compared to the task-positive state. Performing a specific task increases the brain’s energy consumption by less than 5% of the underlying baseline activity. Therefore a large part of the overall activity—from 60 to 80% of all energy used by the brain—occurs in circuits not related to any event of the external world, at the point that different authors (Raichle and Gusnard, 2002; Raichle, 2006; Fox and Raichle, 2007; Zhang and Raichle, 2010) call this intrinsic activity the *brain’s dark energy*, a reference to the unseen energy that also represents the mass of most of the universe. This element draws a parallel between the energy in the universe and the energy of mental life, mostly unconscious and unknown, as claimed by Schopenhauer, Freud, James, and von Helmholtz.

## Default Network Associated With Time and Self

Clinicians have to deal with patients’ past, represented by their medical history and patients’ future, represented by their prognosis (Lingiardi, 2018). Nonetheless, clinicians know that some neuropsychological diseases are mostly stuck in “one specific time,” incapable to perceive different temporal dimensions. For instance, in major depression the patients’ past occupies their mind and thoughts, hijacking their individual existence and preventing the development of their future. Minkowski (1995) described this dimension, claiming that the melancholic patients lack the feeling of time as *propulsive energy*, rather feeling blocked by their attention to the past. A similar scenario, in terms of singular dimensional time, is presented by patients affected by post-traumatic stress disorder, in which they have troubles emerging from the past, a traumatic past that somehow suspended their life, not able to see themselves in the future, with memories intruding into the conscious present in form of flashbacks. In these patients is also common to observe physical symptoms, such as fatigue (Di Francesco et al. under review)^5^ underlining the embodied cognition component and its relationship with emotion and time (Wittmann et al., 2015), symptoms usually accompanied by presence of the timeless dimension of dreams/nightmare.

Another category of individuals with unusual perception of time are the anxious subjects, who show an overestimation of the stimulus durations (Wittmann et al., 2006; Bar-Haim et al., 2010) and report a slowing down of the feeling of time (Lamotte et al., 2014). The psychopathological literature in this field is extensive, including different diagnostic groups and subjective experiences, but the focus of the present manuscript is limited to the “equalization” of past and future at the functional network level of the DN with its possible clinical meanings for the psychodynamic approach. Also, the neuroimaging literature is large on this field, pointing out, among other areas, the key role of the insular cortex, (part of the salience network) in time perception (for meta-analyses, see Lewis and Miall, 2003; Wiener et al., 2010). It is interesting to underline the interactions between insula and the DN, supporting the ability to represent subjects’ bodily states to enable conscious reflection on those states (Molnar-Szakacs and Uddin, 2013; Cieri and Esposito, 2019). The anterior insula constitutes a hub involved in the registration of body sensations and filters external salient events, then sending information to the DN that integrates and elaborates information supporting mental activity connected to the self (Seth and Friston, 2016). Although, the present manuscript is focused on the role of the DN it is worth mentioning that the role of insula (with its connection to the DN) points out the concept that “attention to time” is very connected to “attention to bodily signals” (Wittmann et al., 2015).

In our subjective (“healthy”) experience we *feel* time, clearly distinguishing past from the future within the DN, memories and inferences (as two opposite ways to think about time) seem *entangled*, meaning a lack of independence between states. During the resting state, healthy adult human being can travel through past, present, and future, in a dynamic temporal dimension described as episodic simulation, which makes it possible to project the self and related events into time (i.e., past and future). The projection into time allows the self (and its related events) to detach or decouple itself from the specific point in time and the current environmental context (Northoff, 2011, p. 126).

The DN seems equally active when the subject makes this time travel, thinking about their past or future (Wittmann, 2013; Andrews-Hanna et al., 2014; Esposito et al., 2017; Cieri and Esposito, 2019); this phenomenon is closely related to the formation and growth of the self during development (Tomasello and Rakoczy, 2003; Fransson et al., 2007; Gao et al., 2009). If time and self-share similar neural correlates in terms of functional connectivity within the DN and between the DN and other networks, the investigation of (statistical) correlation/anticorrelation, studies should show some dysfunction parallel with impairment of sense of time and self.

Cognitive neuroscience exploits fMRI to investigate the variability of the brain through the blood oxygen level-dependent (BOLD) resting state signal (Ogawa et al., 1990) as temporal dynamics of neuronal activity. Functional connectivity (FC) is the temporal correlation (statistical dependencies) between spatially remote neurophysiological events (Friston et al., 1993), while atypical FC changes are observed in several neurological and neuropsychiatric disorders, such as mild cognitive impairment, schizophrenia, late life depression, and AD (Buckner, 2013; Andrews-Hanna et al., 2014; Cieri et al., 2017, 2021b, 2022; Esposito et al., 2018; Cera et al., 2019), especially at the level of the DN. Although abnormal functioning of the DN is also clear in individual vulnerability to depression, anxiety, attention deficit, and post-traumatic stress disorders (Slavich and Irwin, 2014), schizophrenia and AD are both specifically considered disconnection syndromes (Geschwind, 1965; Friston and Frith, 1995; Delbeuck et al., 2003; Contreras et al., 2019).

The brain regions that are more active during REST are in the association cortex, including frontal, temporal, and parietal, as well as the retrosplenial cingulate, which shows that during the free associative processes, the association cortices of the human brain communicate to each other easily and without barriers (Andreasen, 2011; Cieri and Esposito, 2018). During the resting state, the neurocognitive awake and healthy system (not affected by disabling neuropsychological disorders) is engaged in the automatic and unconscious registration of bodily, vegetative, endocrine, proprioceptive (internal) inputs and environmental sensorial (external) stimuli, trying to integrate these external and internal stimuli without being overwhelmed and crushed. As mentioned, this activity is mostly bodily, automatic, and unconscious, and, from a temporal perspective it happens in the present, in the *hic et nunc*, the here and now of the subject. During the resting state, the subject is also engaged in a natural free associative dialogue, among emotions, feelings, affects, memories and future plans (Raichle, 2006; Buckner et al., 2008; Northoff, 2011; Christoff et al., 2016; Cieri and Esposito, 2018). The temporospatial dynamics of our brain’s spontaneous activity shapes our mental states, the way we experience ourselves and others in time and space (Spagnolo and Northoff, 2021). This spontaneous activity happens in the individual’s present, with a strict relationship with past and future. These two temporal dimensions are in fact entangled, intertwined, passing from one thought to another in a plastic and fluid way, experiencing a natural flow of thoughts. Consistently with the hypothesis proposed here and with the FEP (Friston et al., 2006; Friston, 2010) some authors (Buckner et al., 2008) define the DN as a *life simulator*, drawing from past experiences, designing and predicting possible future experiences. The DN plays a key role in the hypothesis testing system (Hohwy, 2013), surfing the uncertainty (Clark, 2016), learning from experience (Bion, 1962) through continuous and uninterrupted unconscious and active inferences (Helmholtz, 1866/1962; Friston, 2010). In other words, this network is close to the establishment, development, and growth of a sense of self, to the point that some authors talk about *Default Self* (Northoff and Bermpohl, 2004; Beer, 2007), suggesting a link between this spontaneous activity and the individual sense of self, self-consciousness (Qin and Northoff, 2011; Wittmann, 2013; Davey et al., 2016; Northoff, 2016) or a dynamic sense of self (Spagnolo and Northoff, 2021).

The brain’ topological overlapping between episodic memory and episodic foresight^6^ (Suddendorf, 2006, 2010; Gilbert and Wilson, 2007) was first noticed by Tulving (1985), who showed that an amnesic subject, with no memories of any episodic event, was similarly impaired in imagining events that they may experience in the future. The episodic memory provides experiential material needed to build episodic foresight, in turn essential to learn from experience. According to Bion (1962), the mental growth and the development of the self-depend on the ability of the mind to digest new experiences. We can translate the digestion of new experiences of Bion’s approach in the data assimilation or evidence accumulation under the FEP framework (Cieri and Esposito, 2019).

Freud (1900) in *The two principles of mental functioning* claimed that attention is a psychic function established with the aim of exploring the outside world, such that the data received are already familiar when an inner need arises (p. 120). Bion (1962) in Learning from experience, pointed out how attention is the alpha function (p. 24), where sensory impressions and emotions are transformed into phenomenon, answering to the characteristics required by the thoughts of the dream. The alteration or the inefficacy of the alpha function causes the beta elements to be unmodifiable, elements seen as the ultimate unknowable truth, chaos, the thing-in-itself, according to the Kantian tradition (p. 27). We need sensory organs as instruments of access to the perception of reality to give sense to it, to transform the non-mental elements, sensory impressions (β-elements), in α-elements, through the α-function to give them an emotional value (Bion, 1962). In fact, when the sensorial systems are impaired (for example as a consequence of a pathological aging process), the cognitive system becomes also impaired, with loss of brain entropy (see Cieri et al., 2021a).

There are many clinical examples of amnesic patients with similar impairment in episodic memory and episodic foresight scenes (e.g., Klein et al., 2002a; Hassabis et al., 2007; Squire et al., 2010; Race et al., 2011). A known patient in this area is also one of the most famous cases in cognitive neuroscience, Henry Gustav Molaison (known as HM) suffered from epilepsy, underwent a bilateral medial temporal lobectomy, surgically resecting the anterior two thirds of his hippocampi, parhippocampal cortices, entorhinal cortices, piriform cortices, and amygdalae. This patient was able to recall everything that happened before his lobectomy, but he was unable to remember or learn memories after his surgery to the point that he shook his neuropsychologist’s hand (Brenda Milner), introducing himself with first and last name at any appointment for 40 years, as if it were the first appointment. This patient was equally unable to form episodic memory and episodic foresight. Other works on similar cases confirmed the central role of the hippocampus, underscoring that after bilateral hippocampal damage, patients lose their narrative ability, lacking the power of mental travel either to the past or the future. Their imaginative and planning skills are as strongly impaired as their ability to place themselves into a spatiotemporal context. Instead, they use their logic and semantic knowledge *to create scenarios that might sound fair* (Buzsáki and Llinás, 2017, p. 127).

The episodic memory impairment is the first and most typical symptom in AD and is associated with the similar inability to imagine future scenarios (Addis et al., 2009). In this form of dementia, we observe damages to the hippocampi, parhippocampal, and entorhinal cortices, but also a dysfunction within the DN and between the DN and the other neural networks. In the next paragraph the focus will be the development and decline of the DN, associated with time and self.

## Development and Decline of The Default Network Associated With Time And Self

*“Even a broken clock is right twice a day.”*

(*The Glass Bead Game*, Hesse, 1943).

If episodic memory is correlated to episodic foresight and both are correlated to a sense of self, studies in this field should show a similar ontogenetic increase of memory’s capabilities, with parallel ability to imagine future scenario and a contextual growth of the sense of self. That said, typical diseases in which memory degenerates should be correlated with similar impairment of imagining future scenarios, and both should be associated with the impairment of self.

The idea that episodic memory and episodic foresight are correlated, relying on similar neural structures and functions, was proposed by authors (Tulving, 1985; Szpunar, 2010; Schacter et al., 2012; Schacter and Madore, 2016) able to show that remembering the past and imagining future scenarios share similar cognitive and neural processes. In other words, memory is important for connecting the present with the future (Szpunar et al., 2014).

From an ontogenetic perspective, episodic memory formation appears impossible before the age of 4 (e.g., Tulving et al., 2005). Accordingly, before the third or fourth year of age, children usually have a typically confusing idea of positioning events in both past and future. Ability to simulate future scenarios appears in children after the third or fourth year of life (Gilbert and Wilson, 2007), when the brain organizes its spatiotemporal dynamic in the DN meaning that. In other words the brain areas composing the DN are able to synchronize their activity, associated with the ability of anticorrelation with other networks typically active during tasks, such as the mentioned DAN, active during attention tasks. This negative correlation is in turn associated with children’ ability to retain and recall memories and create new future scenarios. Consistently, the growth of the self is strictly related with the ability to remember and imagine future scenarios together with the growth of the DN in ontogenetic and phylogenetic senses. This network’s connectivity in fact increases through ontogenetic development from birth to adulthood (Fair et al., 2008; Gao et al., 2009). Similarly, the DN regions have undergone significant phylogenetic evolutionary expansion in modern humans (Van Essen and Dierker, 2007). Here the focus is neither the specific building of episodic memory, nor the neural growth of its specific structures (i.e., hippocampus), but the relationship between past and future, with its connection with a sense of self. From a neural perspective, the focus is on functional connectivity of the DN and its relationship with other neural networks, particularly the DAN.

In a modern healthy adult awake neurocognitive system, the DN and the DAN should show a negative correlation, both during rest and tasks. During rest the DN should be more active, and the DAN should be more silent. During attention tasks the opposite should be true. Consistently, greater DN-DAN negative correlation is associated with higher cognitive control and better working memory performance (Anticevic et al., 2012; Chai et al., 2012).

**FIGURE 2:**
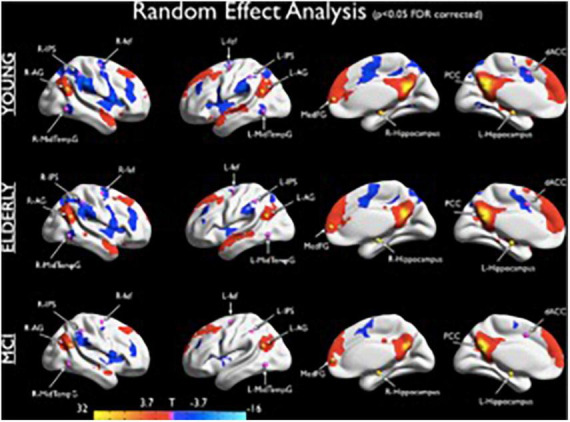
Seed based connectivity maps (seed: PCC) obtained from the random effects group analyses showing the DMN and the anticorrelated DAN for the three groups. The statistical maps were thresholded at *p* < 0.05 (corrected for multiple comparisons using FDR) and superimposed on a partially inflated Talairach template (from Esposito et al., 2018).

The self and the DN-DAN anticorrelation grow together during individual development, in conjunction with a more coherent and solid sense of time and self. In fact, parallel with ontogenetic and phylogenetic function and evolution of the self, this anticorrelation appears during the first year of life, strengthens during the second year (Barber et al., 2013), when the child begins to recognize themself as an object, and becomes stronger in adults to support the development of executive functions and working memory from childhood to adulthood (Andrews-Hanna et al., 2007). The DN-DAN negative correlation seems to show an “inverted-U” shape, increasing during development and decreasing during physiological (Wu et al., 2011), and, especially, cognitively pathological aging (Cieri et al., 2021a). Given this evidence, it is reasonable to observe a decline of this anticorrelation during mild cognitive impairment (Esposito et al., 2018; Figure 2) and for it to be even more solid during the AD (Kundu et al., 2019), representing a possible biomarker of neuroaging and cognitive decline.

Late onset AD is the most common cause of dementia; a global health challenge with huge impact in size and costs. In 2021, 6.2 million Americans aged 65 and older living with AD, Alzheimer’s Association (2022). Globally, the numbers of people living with dementia will increase from 50 million in 2018 to 152 million in 2050, a 204% increase (Prince et al., 2016). This form of dementia is a complex biopsychosocial disease in which biopsychosocial factors interact in a dynamic and complex way. In this field, there are potential brain imaging biomarkers investigable with different imaging approaches, such as the fMRI.

Although there are different signs, symptoms, and possible biomarkers of AD, one of the first and most disabling neuropsychological signs in these patients is the impairment of episodic memory, where the access to recent events is tragically impaired. Clinicians are more interested in the investigation of the past (memory) of the patients, compared to their planning ability (future), but these temporal dimensions are correlated, lying on the same structure (hippocampus) and the same neural network (the DN). As we have seen, this proposal of a close relationship between time and self at the level of the DN, has shown intriguing parallel among episodic memory, episodic foresight, and a parallel maturation of a sense of self. Still, the evidence should show some changes/impairment with pathological aging, when the subject can suffer a loss of cognitive abilities that can begin with episodic memory loss, as in the case of AD. In this case, the resting state-fMRI studies should show some changes at the DN level.

The posterior DN, such as the posterior cingulate cortex and the precuneus, supports autobiographical memory, episodic memory retrieval, future planning, records of bodily sensations, self-reported mental processes, and monitoring psychological states (Borsook et al., 2015; Cieri and Esposito, 2019; Cieri et al., 2021b,^2022^). During AD, this brain area is the first affected by atrophy and amyloid-β deposition (Greicius et al., 2003; Buckner et al., 2009). From a functional perspective, we observe a disease progression severity correlated with reduced FC, especially at the level of the posterior DN, as compared to age-matched controls (Zhang et al., 2010; Zhou et al., 2010). According to the hypothesis proposed, the posterior DN has a strategic role in coordinating the interactions among different sensory areas and frames of reference concerning the internal (body) and the external (environment). In fact, bilateral lesions to these areas are related to a virtual breakdown of information integration in the thalamocortical system (Tononi, 2004). AD patients show a similar difficulty in remembering and imagining future scenarios, slipping into a fragmented present without past or future. AD is in fact marked by an inexorable loss of self’ functions. In this sense, Proust and Sturrock (2003) words sound extremely appropriate, when he claimed that reality is shaped only in the memory. Despite the concept of self, with its complex functions, is not enclosed or circumscribed in a structure (or a network such as the DN), or in a function (such as memory, see Klein et al., 2002b), in losing their memory, these patients seem to lose their reality, their self.

AD is not the only disorder able to show this phenomenon; other patient populations show episodic memory and episodic foresight impairments, including patients with temporary amnesia (Juskenaite et al., 2014), depression (Williams et al., 1996), schizophrenia (D’Argembeau et al., 2008), and post-traumatic stress disorder (Brown et al., 2014).

Lack of memory overlaps with lack of prospect, planning, and imagining future scenarios. This absence of past and future is associated with a loss of reality, therefore a *loss of self*. The function of the DN is related to the self, supporting the internal mental simulations used in adaptive ways (Andrews-Hanna, 2012; Buckner, 2013), consistently with the FEP, in which the system is engaged with simulations, searching of patterns, trying to maintain an internal sensitive balance of the organism, and supporting internal mental simulations used adaptively. In other words, the neurocognitive system is constantly engaged in the search of patterns, trying to build and update its model of the world. In this conscious and unconscious engagement, the DN has a substantial role, mediating between the external and internal stimuli, building dynamic mental future simulations based on past personal experiences. In AD we observe a fall of the individual’s time that leaves the patients stuck in a slavery of the present, losing their self’ functions.

According to Schacter and Addis (2007):

For more than 100 years, memory has been the object of experimental studies that have focused almost exclusively on its role in preserving and recovering the past. We think it is time to try to understand some of memory’s errors by looking to the future (p. 27).

It might be also time to try to understand the relationship between time and self with an approach able to integrate the subjectivity of psychoanalysis with methods of neuroscience, such as the resting state fMRI.

## Discussion

“*It’s a poor sort of memory that only works backwards,”*

*the Queen remarked.*

(*Through the Looking Glass;* Carroll, 1920).

The DN’s processes has been proposed as close to the mediation function attributed by Freud to the Ego, where the system attempts to integrate and elaborate the internal and external stimuli, with the aim of lowering the brain entropy (Carhart-Harris and Friston, 2010; Carhart-Harris et al., 2014; Cieri and Esposito, 2019; Cieri et al., 2021a). The studies described are consistent with the ideas of a proximity between time and self, and from a neuro-functional point of view, perception of time and sense of self share the same neural network: the DN, associated with our *stream of consciousness*. This can have important potential theoretical and clinical significance for psychoanalysis and psychodynamic neuroscience. From a psychodynamic view, the DN might be seen as a sort of neural configuration of the *free floating attention* proposed by Freud (1922), necessary to the psychoanalyst to be in the right “disposition” to listen and feel the stream of consciousness of the patient, allowing the free associative process in both participants of the analytic setting. The free floating attention, together with the free association, are operated by the self, continuously and constantly built and established over time. Both free floating attention and free associative process are *timeless* state, meaning that they happen in the present, but they are both about past and future, strongly associated to the DN’s activity.

Freud (1922) claimed:

[The doctor] should withhold all conscious influences from his capacity to attend and give himself over completely to his “unconscious memory.” The doctor must put himself in a position to make use of everything he is told for the purposes of interpretation and of recognizing the concealed unconscious material without substituting a censorship of his own for the selection that the patient has forgone (p. 111–112).

In 1922, Freud added that the attitude of the clinician should surrender to the unconscious *free floating attention*, also called *evenly suspended attention*, avoiding construction of explicit and conscious expectations (p. 239). A couple of years later (1925), he returned to the topic in his correspondence with Binswanger, specifying that the unconscious must be replaced with the preconscious (Reppen, 2003), a statement that seems to come even closer to the DN’s activity. In this scenario, the *free floating attention*, based on the DN, can stimulate the free associative method in both participants–to the point that we might speak about a *default communication*, *default relationship*, or *default therapy* meaning a communication that would seem to involve the DN in a peculiar way, where the history of the individual, their past, present and future narrative, overlap in the same neural process. This can find a symbolic representation in the transferral and controtransferral processes of the psychoanalytic treatment, where the transference can be seen as the unconscious repetition in the here and now of pathogenic conflicts from the past (Kernberg, 2016).

Freud (1933) pointed out the *timeless* of unconscious, writing about the unconscious instance of the id, he claimed that in the id, there is nothing close to the idea of time (p. 106). This idea finds an intriguing overlap with the DN. If we consider the self as composed of the id, the ego, and the superego, the id could intuitively be seen as *the most timeless*. Nevertheless, the self is formed through the biopsychosocial history of the individual, built and place in time.

In the current theoretical framework, both episodic memory and episodic foresight rely on neurocognitive resources at the DN, suggesting that the link between past and future might have clinical implications, from a neuropsychological and neuroscientific perspective. An important challenge for future research should be the more accurate neurocognitive characterization of episodic memory and episodic foresight, exploring more specifically their common, or different, neural processes. Associating the neuropsychological investigation with a resting state fMRI, would allow a deeper knowledge on neural correlates of both mental processes. This approach can yield important information about our perception of time, our self, and the psychopathological expressions in which these dimensions are impaired, such as AD. Furthermore, the narrative of the psychoanalytic patient could help a process of subjectivity too often missing in the current *mindless* neuroscientific approach. The subjectivity that emerges in the psychodynamic therapeutic relationship could provide useful insights in the study of the perception of time and self.

The importance of *learning from experience* has a tremendous value in terms of human survival, adaptation, and evolution, both as individuals and a species. In this scenario it is crucial to consider the affective environment, the unconscious manifestations, attachment, and relationships with their key role defining the individual’s self in time.

Psychoanalysis works with uncertainty and entropy, being a witness and sometimes a guardian (at least of the desire’s) chaos. As a patient with an obsessive personality structure, after years of therapy, once wisely said: “I live with the certainty of my uncertainty.” In this patient a neurological condition happened during psychoanalytic psychotherapy, changing in intriguing way the perception of time together with the patient’s self.

## Data Availability Statement

The original contributions presented in this study are included in the article/supplementary material, further inquiries can be directed to the corresponding author/s.

## Author Contributions

FC contributed to the conceptualization, formal analysis, investigation, methodology, visualization, writing—original draft, writing review, and editing.

## Conflict of Interest

The author declares that the research was conducted in the absence of any commercial or financial relationships that could be construed as a potential conflict of interest.

## Publisher’s Note

All claims expressed in this article are solely those of the authors and do not necessarily represent those of their affiliated organizations, or those of the publisher, the editors and the reviewers. Any product that may be evaluated in this article, or claim that may be made by its manufacturer, is not guaranteed or endorsed by the publisher.

## Footnotes

1 The idea of complementarity comes from James (1890) who coined the term, imported by Bohr in the realm of physics, originally with the purpose of replacing the term “wave–particle duality,” in his “Como Lecture” in 1927 (Bohr, 1928, p. 566).

2 In this field, the “experiment” by King Frederick II of Sicily (thirteenth century), who had an interest discovering the natural language of humans, is also sadly known. His question was: if no one speaks with the babies, what language will they learn? The king requested for some children in his kingdom, raising them in total isolation, with no affective relationships, (no attachment), for some years, allowing only specific indicated persons to silently run and leave food for them, with no interactions. Not able to show the “real natural language of human being,” all the children in this “experiment” died from an extreme case stress exposure (Sapolsky, 1994).

3 Also, the description of the FEP is beyond the scope of this manuscript. This principle, even in its relationship with psychoanalysis, has been presented in other works (Solms and Friston, 2018; Cieri and Esposito, 2019; Rabeyron, 2021; Solms, 2021).

4 Although in psychoanalysis the Self and the Ego are two distinct concepts, where the Ego is one part of the self, this manuscript does not always distinguish between these two instances because from a neuroscientific perspective, their differentiation would be challenging and beyond the scope of this manuscript.

5 Di Francesco, G., Cieri, F., Sciarra, P., Ballarini, V., Di Ianni, M., and Santarone, S. (under review). Fatigue as mediator factor in PTSD patients after allogeneic hematopoietic stem cell transplantation. *Psychol. Trauma Theory Res. Pract. Policy*

6 Episodic foresight is a term proposed by Suddendorf (2006, 2010) to define the ability to imagine future scenarios. Other synonyms are episodic future thinking (Atance and O’Neill, 2001), envisioning the future (Buckner and Carroll, 2007), episodic simulation of future events (Schacter et al., 2007), and mental time travel into the future (Suddendorf and Corballis, 1997).
